# Lack of Cross-protection against *Bordetella holmesii* after Pertussis Vaccination

**DOI:** 10.3201/eid1811.111544

**Published:** 2012-11

**Authors:** Xuqing Zhang, Laura S. Weyrich, Jennie S. Lavine, Alexia T. Karanikas, Eric T. Harvill

**Affiliations:** The Pennsylvania State University, University Park, Pennsylvania, USA

**Keywords:** Bordetella holmesii, Bordetella pertussis, Bordetella, vaccines, antibody, immune evasion, bacteria, vaccination, immunization, Massachusetts, United States, *Suggested citation for this article*: Zhang X, Weyrich LS, Lavine JS, Karanikas AT, Harvill ET. Lack of cross-protection against *Bordetella holmesii* after pertussis vaccination. Emerg Infect Dis [Internet]. 2012 Nov [*date cited*]. http://dx.doi.org/10.3201/eid1811.111544

## Abstract

*Bordetella holmesii*, a species closely related to *B. pertussis,* has been reported sporadically as a cause of whooping cough–like symptoms. To investigate whether *B. pertussis*–induced immunity is protective against infection with *B. holmesii*, we conducted an analysis using 11 human respiratory *B. holmesii* isolates collected during 2005–2009 from a highly *B. pertussis*–vaccinated population in Massachusetts. Neither whole-cell (wP) nor acellular (aP) *B. pertussis* vaccination conferred protection against these *B. holmesii* isolates in mice. Although T-cell responses induced by wP or aP cross-reacted with *B. holmesii*, vaccine-induced antibodies failed to efficiently bind *B. holmesii*. *B. holmesii*–specific antibodies provided in addition to wP were sufficient to rapidly reduce *B. holmesii* numbers in mouse lungs. Our findings suggest the established presence of *B. holmesii* in Massachusetts and that failure to induce cross-reactive antibodies may explain poor vaccine-induced cross-protection.

## Abstract 2

Vaccines for *B. pertussis* do not protect against circulating strains of a closely related respiratory pathogen.

*Bordetella pertussis* and *B. parapertussis* commonly cause whooping cough, a highly contagious, acute coughing illness, in humans (*1*,*2*). Licensed in the mid-1940s, the first whooping cough vaccines consisted of whole-cell inactivated *B. pertussis* (wP), and their use led to a dramatic decrease in disease incidence by the mid-1960s (*2*,*3*). The potential for health risks related to wP vaccine, however, led to adaptation of acellular (aP) vaccines, which contain combinations of purified *B. pertussis* proteins. Despite high vaccine coverage, reported whooping cough incidence in industrialized countries has been increasing during the past 20 years, although this could be the result of greater awareness and improved analytical tools (*4*–*9*).

In November 1983, the Centers for Disease Control and Prevention (CDC) received a gram-negative bacterium isolated from an asplenic patient (*10*). During the following decade, additional clinical isolates with the same microbiological characteristics (slow-growing, gram-negative, small coccoid, asaccharolytic, oxidase negative, nonmotile and brown-soluble-pigment-producing) were submitted to the CDC for identification (*10*). Subsequent biochemical analysis, 16S rRNA sequencing, and DNA relatedness studies revealed that these strains were new *Bordetella* species, which was named *Bordetella holmesii* to honor Barry Holmes (*10*). Since then, this bacterium has been isolated from numerous countries, including Australia, Canada, Chile, France, Germany, Japan, Netherlands, Switzerland, the United Kingdom, and the United States (*6*–*15*). These findings indicate that *B. holmesii* is a widespread pathogen among populations that are highly vaccinated against *B. pertussis*.

Comparative analysis of *B. holmesii* and *B. pertussis* by using 16S rRNA suggests that *B. holmesii* is closely related to *B. pertussis*, but further analysis of cellular fatty acid composition, housekeeping genes, and the BvgAS locus suggests that *B. holmesii* may not share many of the highly conserved virulence factors of *B. pertussis* (*16*). Antibodies against *B. pertussis* pertactin, pertussis toxin, fimbriae, adenylate cyclase toxin, and filamentous hemagglutinin recognize few, if any, proteins from multiple *B. holmesii* isolates (*14*), results that suggest *B. holmesii* may be antigenically distinct from *B. pertussis*.

Although *B. holmesii* has been isolated primarily from immunocompromised hosts (asplenic or sickle cell disease patients and transplant recipients) (*14*,*17*–*20*) and was first isolated from blood, the bacterium has also been found to cause respiratory diseases (*11*,*12*,*21*–*23*). *B. holmesii* was isolated from pleural fluid and lung biopsy specimens from an immunocompetent adolescent who had fever and pulmonary fibrosis (*12*) and from sputum of patients with respiratory failure (*22*). Moreover, *B. holmesii* was isolated from nasopharyngeal specimens of previously healthy persons who had whooping cough–like symptoms, including paroxysms, whooping, or post-tussive vomiting (*11*,*21*,*23*). Therefore, *B. holmesii* appears to be able to colonize the respiratory tract in the same manner as other *Bordetella* species. A case study in Japan also found epidemiologic links between 5 persons colonized with *B. holmesii*, which indicates the ability of this pathogen to transmit from person to person (*6*).

In collaboration with the Massachusetts Department of Public Health (MDPH), we reviewed *B. holmesii* surveillance data collected in Massachusetts during 2005–2009. *B. holmesii* was isolated from several patients experiencing whooping cough–like symptoms. By using a murine infection model, we examined the effects of *B. pertussis* vaccination on *B. holmesii* infection susceptibility.

## Materials and Methods

### Identification of *B. holmesii* Cases in Massachusetts

Culture-confirmed *B. holmesii* cases identified during 2005–2009 by the State Laboratory Institute at the MDPH were included in our analysis. According to MDPH guidelines, a nasopharyngeal swab was cultured if the patient was <11 years of age or had a cough for <14 days. For all other patients (>11 years of age and >14 days of cough), a serum test was performed. Details on culturing methods and *Bordetella* spp. identification tests performed have been described (*21*). A total of 41 *B. holmesii* infections were reported; the case records, including symptomology for 26 of these, are maintained in the Massachusetts Virtual Epidemiologic Network.

### Bacterial Strains and Growth

*B. pertussis* strain 536 (*24*) and *B. parapertussis* strain CN2591 (*25*) have been described. *B. holmesii* strain P3421 was isolated in Massachusetts and used for animal experiments. Bacteria were maintained on Bordet-Gengou agar (Difco, Sparks, MD, USA) supplemented with 10% sheep’s blood (Hema Resources, Aurora, OR, USA) without antimicrobial drugs (*B. holmesii*) or with 20 µg/mL streptomycin (Sigma-Aldrich, St. Louis, MO, USA) (*B. pertussis* or *B. parapertussis*). Liquid cultures were grown overnight in Stainer-Scholte broth at 37°C to mid-log phase (*26*,*27*).

### Phylogenetic Analysis

Phylogenetic analyses were performed on the basis of *atpD, rpoB, tuf,* and *rnpB* gene sequences as described (*16*). Gene amplifications were completed on 30 *B. holmesii* isolates obtained from MDPH or the CDC, *B. pertussis* strain 536, *B. parapertussis* strain 2591, *B. bronchiseptica* strain RB50, *B. avium* strain 197N, and *B. hinzii* strain BC304. Concatenated sequences were aligned and used to construct unweighted pair group method using average linkages trees in MEGA4 software (www.megasoftware.net/mega4/mega.html).

### Animal Experiments

All protocols were approved by Institutional Animal Care and Use Committee (IACUC). All animals were C57BL/6 mice and were handled in accordance with institutional guidelines (IACUC approval no. 31297). Animal experiments were performed as described, with 4 mice per group, and were performed in replicate (*28*–*33*). For vaccinations, sedated 4–6-week-old mice were vaccinated by intraperitoneal injection of 10^8^ CFU of heat-inactivated (65°C for 30 min) bacteria in 200 µL of phosphate-buffered saline (PBS; Omnipur, Gibbstown, NJ, USA) for whole-cell *B. holmesii* vaccine (wH) or wP *B. pertussis* vaccine; one fifth human dose of Adacel (Sanofi-Pasteur, Swiftwater, PA, USA) (0.5 µg PT, 1 µg FHA, 0.6 µg pertactin, 5 µg fimbriae 2 and 3 per mouse) with Imject Alum (Thermo Scientific) (aP); or only Imject Alum in 200 µL PBS on days 14 and 28 before challenge (*28*,*33*).

For challenge, 50 µL PBS containing 5 × 10^5^ CFU of *B. pertussis* or *B. parapertussis* or 10^7^ CFU of *B. holmesii* was added by pipetting onto the external nares of sedated mice (*29*). A larger inoculum of *B. holmesii* was used to achieve reproducibility and detect *B. holmesii* from the respiratory tract at later time points, because it is cleared more rapidly from the lower respiratory tract than are *B. pertussis* or *B. parapertussis*. For adoptive transfer of serum antibodies, mice were vaccinated with the indicated bacteria on days 0 and 14, and serum samples were collected on day 28 from vaccinated or naive animals. Serum samples of 200 µL were intraperitoneally injected immediately before mice were inoculated with 5 × 10^6^ CFU of *B. holmesii* (*30*,*32*). Bacterial numbers were quantified as described (*30*).

### Splenocyte Restimulations

Splenocytes were isolated from vaccinated mice as described (*31*,*33*) and stimulated with either media alone or media containing 10^7^ CFU (multiplicities of infection of 5) of the indicated heat-killed bacteria (*28*,*31*). After 3 days, the supernatants were collected and analyzed for interferon (IFN) γ and interleukin-10 (IL-10) production by using ELISA (R&D Systems, Minneapolis, MN, USA) according to the manufacturer’s instructions.

### Titer ELISAs

Antibody titers were determined as described (*34*–*36*). In brief, wP- or wH-induced/naive serum samples (1:200 dilution) or aP/adjuvant-induced serum samples (1:50 dilution) from each individual mouse were added to and serially diluted 1:2 across plates coated with heat-inactivated exponential-phase bacteria. After incubation, samples were probed with 1:4,000 dilution of goat anti-mouse Ig horseradish peroxidase–conjugated antibodies (Southern Biotech, Birmingham, AL, USA). Titers were determined by using the endpoint method (*33*).

### Western Blot Analysis

Lysates containing 10^7^ CFU of indicated heat-killed bacteria were subjected to 10% sodium dodecyl sulfate–polyacrylamide gel electrophoresis under denaturing conditions. Polyvinylidene fluoride membranes (Millipore, Billerica, MA, USA) were probed overnight with either naive serum (1:100 dilution) or wH- (1:500 dilution), wP- (1:500 dilution), aP- (1:100 dilution) induced serum. A 1:10,000 dilution of goat anti-mouse Ig horseradish peroxidase–conjugated antibodies was used as the detector antibody (*35*,*37*). The membrane was visualized with ECL Western Blotting Detection Reagent (Pierce Biotechnology, Rockford, IL, USA).

### Statistical Analysis

Mean ± SE values were determined for all appropriate data. Two-tailed, unpaired Student *t* tests, analysis of variance and Tukey’s simultaneous test in Minitab (www.minitab.com) with similar significance were used to determine statistical significance between groups.

## Results

### *B. holmesii* Endemicity in Massachusetts

**Figure 1:**
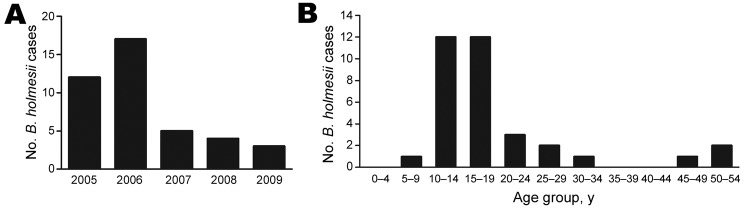
*Bordetella holmesii* cases in Massachusetts, USA. A) Nasopharyngeal specimens culture-positive for *B. holmesii* infection as confirmed by the Massachusetts Department of Public Health, by year, 2005–2009. B) Age distribution of case-patients with *B. holmesii* infection during 2005–2007 (cases shown in the Table).

In 1999, Yih et al. reported an increase in culture-positive *B. holmesii* cases from 1995 to 1998 (0.2% to 0.6%) (*23*). Here, collaborating with the same MDPH research team, we report the numbers of *B. holmesii* culture-positive nasopharyngeal specimens submitted to the MDPH during 2005–2009. Over these 5 years, *B. holmesii* was isolated from the nasopharyngeal swabs of 41 patients who had similar respiratory symptoms, which is 8 more total cases than observed by Yih et al. during 1994–1998 (33 total cases) (*23*). At least 2 isolates were recovered each year, and 17 cases were identified in 2006, the highest number observed (Figure 1, panel A). The rate of *B. holmesii*–positive nasopharyngeal swabs ranged from 0.1% to 0.4%, in line with previous results (Table). Similar to observations made in prior years, 71% of cases occurred in persons 10–19 years of age (Figure 1, panel B), compared with >80% of cases during 1994–1998.

**Table:** Results of testing of nasopharyngeal swabs for each *Bordatella* species at Massachusetts State Laboratory Institute, 2005–2007*

Test results and species detected	No. (%) swabs		
	2005	2006	2007
Positive			
* B. holmesii*	12 (0.37)	17 (0.35)	5 (0.14)
* B. parapertussis*	23 (0.71)	14 (0.29)	17 (0.49)
* B. pertussis*	196 (6.05)	188 (3.87)	204 (5.87)
Negative	3,007	4,644	3,248
Total tested	3,238	4,863	3,474

*Years for which full data on all laboratory specimens tested were available.

Symptom documentation was obtained for 26 of the 41 cases. All 26 of these patients had a cough; 17 (65%) had a paroxysmal cough, 6 (23%) had post-tussive vomiting, and 4 (15%) had an inspiratory whoop. Nineteen patients (73%) exhibited >1 of these classic symptoms of whooping cough and met the World Health Organization clinical case definition for pertussis (www.who.int/immunization_monitoring/diseases/pertussis_surveillance/en/index.html). No data were collected regarding any previous underlying diseases or potential co-infections among these patients. Although we have no evidence that *B. holmesii* is the causative agent of these pertussis-like illness, these data suggest that *B. holmesii* is consistently present in the nasopharynx of a small number of patients who have respiratory infections in Massachusetts.

### Phylogenetic Relationships among *B. holmesii* Isolates

**Figure 2:**
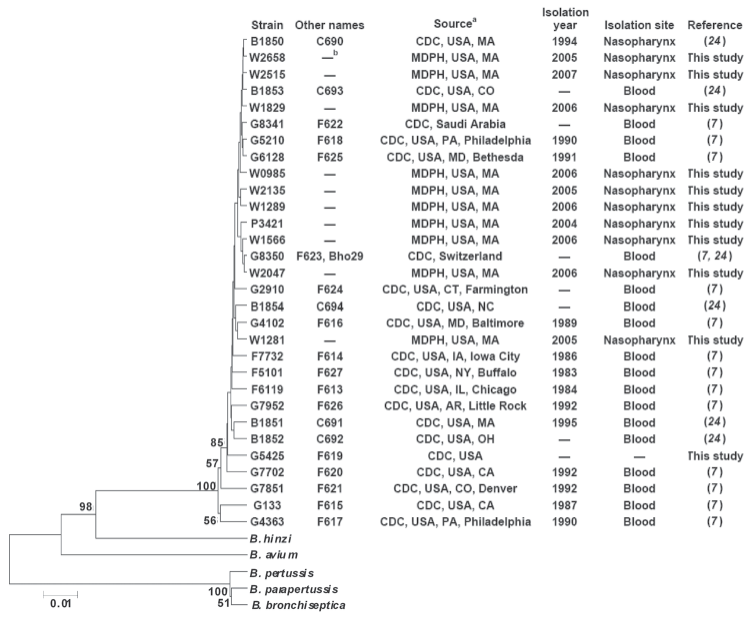
Phylogenetic tree showing 30 *Bordatella holmesii* isolates, *B. pertussis* 536, *B. parapertussis* 2591, *B. bronchiseptica* RB50, *B. avium* 197N, and *B. hinzii* BC304. Tree was constructed on the basis of concatenated nucleotide sequences of *atpD*, *rpoB*, *tuf* and *rnpB* genes. Bootstrap values >50% in 1,000 replicates are indicated. Scale bar indicates substitutions per site. CDC, Centers for Disease Control and Prevention; MDPH, Massachusetts Department of Public Health; –, unknown. Scale bar indicates nucleotide substitutions per site.

To evaluate the phylogenetic relationships among *B. holmesii* isolates, an unweighted pair group method using average linkages tree was constructed on the basis of concatenated nucleotide sequences amplified from regions of *atpD*, *rpoB*, *tuf*, and *rnpB* genes (*16*). Twelve isolates were obtained from CDC (designated with the letter ‘G’), while the remaining isolates were obtained from the MDPH. Consistent with previous findings (*16*), all the *B. holmesii* isolates tested were more closely related to *B. hinzii* and *B. avium* than to the classical bordetellae (Figure 2). Although single-nucleotide polymorphisms exist among *B. holmesii* isolates, pairwise comparisons among the 10 nasopharyngeal isolates from Massachusetts showed >99% sequence identity among 2,958 aligned bases, indicating their close relatedness. By comparison, the CDC isolates were more diverse, with the lowest percent sequence identity 97.5% between CDC strains G7851 and G4363. Moreover, *B. holmesii* isolates from blood and nasopharyngeal specimens do not cluster separately, which suggests that evolutionary relationship among *B. holmesii* isolates is not associated with the anatomic site of isolation.

### *B. holmesii* Susceptibility to *B. pertussis* Vaccine-induced Immunity

Most records for the identified *B. holmesii* culture-positive cases did not include information regarding vaccination history; however, 16 patients that were culture positive for *B. holmesii* received >3 doses of pertussis vaccine. The 2010 coverage among children in Massachusetts for DTaP (diphtheria toxoid–tetanus toxoid–acellular pertussis vaccine) 4-dose vaccine was estimated to be >91%, ranking third among the 50 United States (www.cdc.gov/vaccines/stats-surv/imz-coverage.htm). However, adolescents and adults have lower *B. pertussis* vaccine coverage and waning immunity against *B. pertussis*. More adolescents and adults compared with children were culture-positive for *B. holmesii* in Massachusetts, which suggests that *B. pertussis* vaccine may confer some level of protection against *B. holmesii* in recently vaccinated persons, similar to the cross-protection against *B. bronchiseptica* in a murine model of infection (*38*). Although DTaP was not designed to prevent *B. holmesii* infections, it is critical to evaluate whether *B. pertussis* vaccines confer cross-protection against *B. holmesii*.

**Figure 3:**
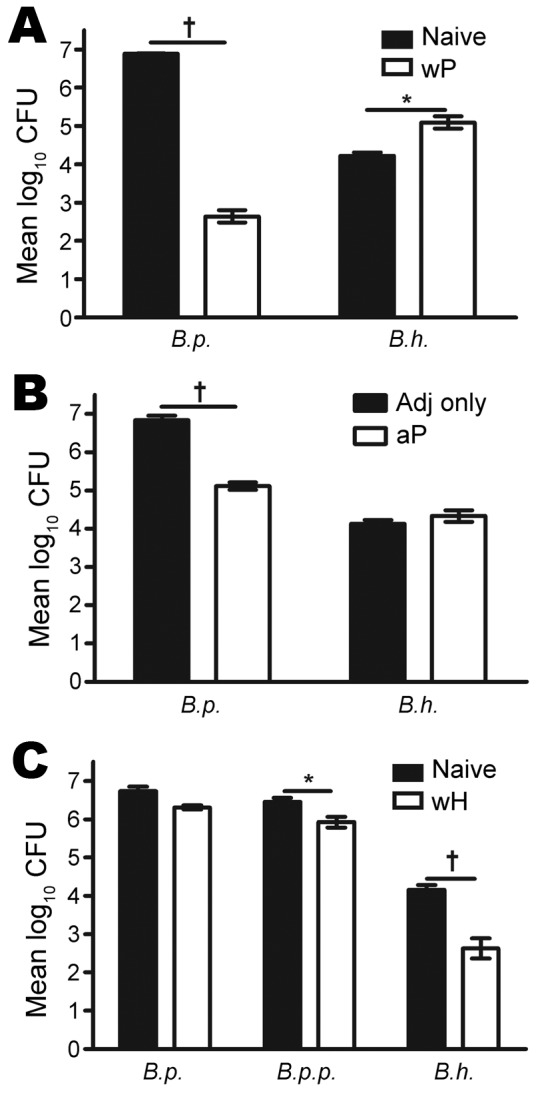
Results of testing of *Bordatella pertussis* and *B. holmesii* vaccines and protection against *B. holmesii* in mice. A) Mice vaccinated with whole-cell pertussis vaccine (wP) versus naive mice; B) mice vaccinated with aceullar pertussis vaccine (aP) versus adjuvant (adj) only vaccinated; C) mice vaccinated with whole-cell *B. holmesii* vaccine (wH) versus naive mice. All mice were challenged with *B. pertussis* (*B.p.*), *B. parapertussis* (*B.p.p.*) or *B. holmesii* (*B.h.*), and euthanized on day 3 postinoculation. Error bars indicate SE. *p≤0.05; †p≤0.01.

To test whether *B. pertussis* vaccines provide cross-protection against *B. holmesii*, we vaccinated C57BL/6 mice with wP or aP. These mice or vaccine-naive mice were then challenged with *B. holmesii* or *B. pertussis* and euthanized 3 days later. Because *B. holmesii* colonization efficiency in the murine respiratory tract is low compared with the classical bordetellae, possibly because of the decreased attachment to mouse respiratory epithelium (A.T. Karanikas and E. T. Harvill, unpub. data), a higher challenge dose was used than for *B. pertussis*. wP vaccination reduced *B. pertussis* numbers in the lungs by >99.99% compared with naive mice; however, wP failed to reduce *B. holmesii* numbers (Figure 3, panel A). In fact, wP vaccination appeared to increase *B. holmesii* numbers in the lungs compared with naive mice. Although aP-vaccinated mice also reduced *B. pertussis* numbers in their lungs by ≈98% (Figure 3, panel B), they were not capable of reducing *B. holmesii* numbers. Together, these data indicate that wP- or aP-induced immunity does not protect against *B. holmesii* infections.

To determine whether *B. holmesii* immunization induces protection against itself or cross-protection against *B. pertussis* and/or *B. parapertussis*, we vaccinated C57BL/6 mice with heat-killed *B. holmesii* (wH) and challenged these or naive mice with *B. pertussis*, *B. parapertussis*, or *B. holmesii*; the mice were then euthanized on day 3 postinoculation for bacterial quantification. Similar numbers of *B. pertussis* were recovered from the lungs of naive and wH-vaccinated mice (Figure 3, panel C), an indication that *B. holmesii* vaccination failed to reduce *B. pertussis* numbers within 3 days. However, *B. holmesii* vaccination reduced the *B. parapertussis* load by ≈70% in the lungs, which indicates a modest cross-protection provided by *B. holmesii* against *B. parapertussis*. Compared with naive mice, mice who received *B. holmesii* vaccination had a ≈97% reduction of *B. holmesii* in the lungs (Figure 3, panel A). This experiment indicates that *B. holmesii* antigens induce efficient protective immunity against *B. holmesii* but have little effect against *B. parapertussis* and are even less protective against *B. pertussis*.

### T-cell Responses to wH and wP/aP for *B. holmesii*

**Figure 4:**
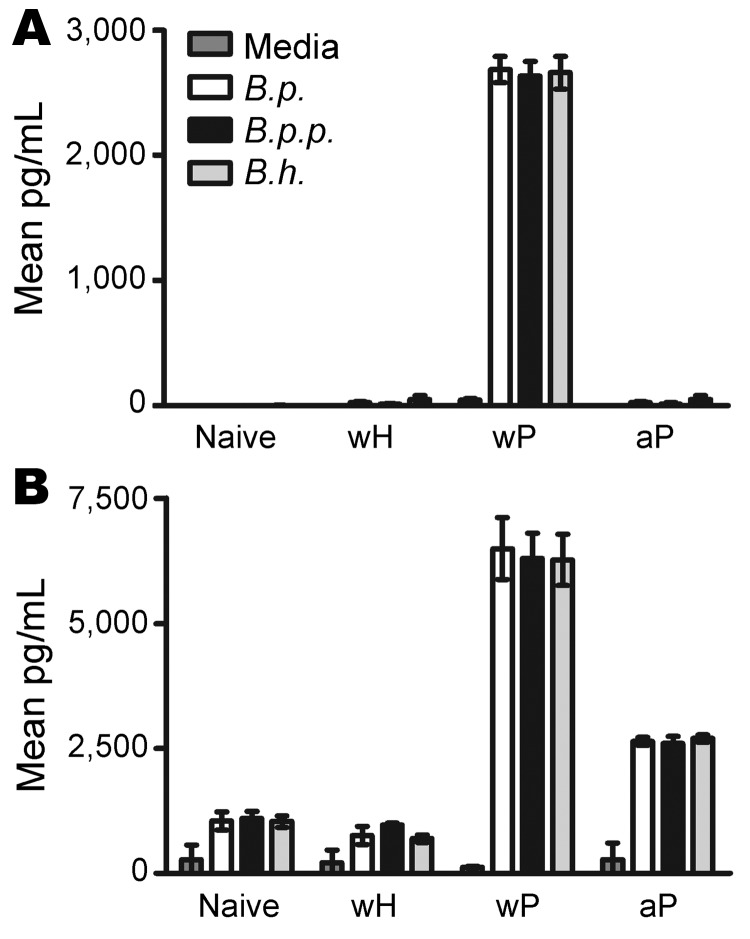
Comparison of splenic interferon (IFN)–γ (A) and interleukin (IL)–10 (B) responses in naive mice versus mice vaccinated with whole-cell *Bordetella holmesii* vaccine (wH), whole-cell pertussis vaccine (wP), and acelullar pertussis vaccine (aP). Splenocytes from naive mice or wH-, wP-, or aP-vaccinated mice were stimulated with media only or media containing heat-killed *B. pertussis* (*B.p.*), *B. parapertussis* (*B.p.p.*), or *B. holmesii* (*B.h.*). Error bars indicate SE.

Because wP and aP fail to reduce *B. holmesii* numbers, we hypothesized that the pertussis vaccines may induce a different T-cell response than the wH vaccine does. To determine whether T-cell responses after vaccination are cross-reactive, splenocytes from naive or wH-, wP-, or aP-vaccinated mice were stimulated with media or heat-killed *B. pertussis*, *B. holmesii*, or *B. parapertussis* for 72 hours. Cell culture supernatant cytokine concentrations of IFN-γ and IL-10, representing Th1 and Treg cytokines, respectively, were determined. wH vaccination did not induce IFN-γ and IL-10 production by splenocytes at greater levels than those produced by naive splenocytes (Figure 4). However, splenocytes from wP-vaccinated mice produced high levels of IFN-γ and IL-10 on stimulation with heat-killed *B. pertussis*, *B. parapertussis*, or *B. holmesii*, which indicates that wP induces a strong cross-reactive T-cell response to *B. holmesii*. Although splenocytes from aP-vaccinated mice produced little IFN-γ, they produced ≈2,500 pg/mL IL-10 on stimulation with heat-killed *B. pertussis*, *B. holmesii*, or *B. parapertussis*, which indicates cross-reactive T-cell responses following aP vaccination. These data indicate that *B. pertussis* vaccines can induce cross-reactive T-cell responses to *B. holmesii* and thus do not explain the lack of cross-protection between these species.

### wP/aP-induced Antibodies for *B. holmesii*

**Figure 5:**
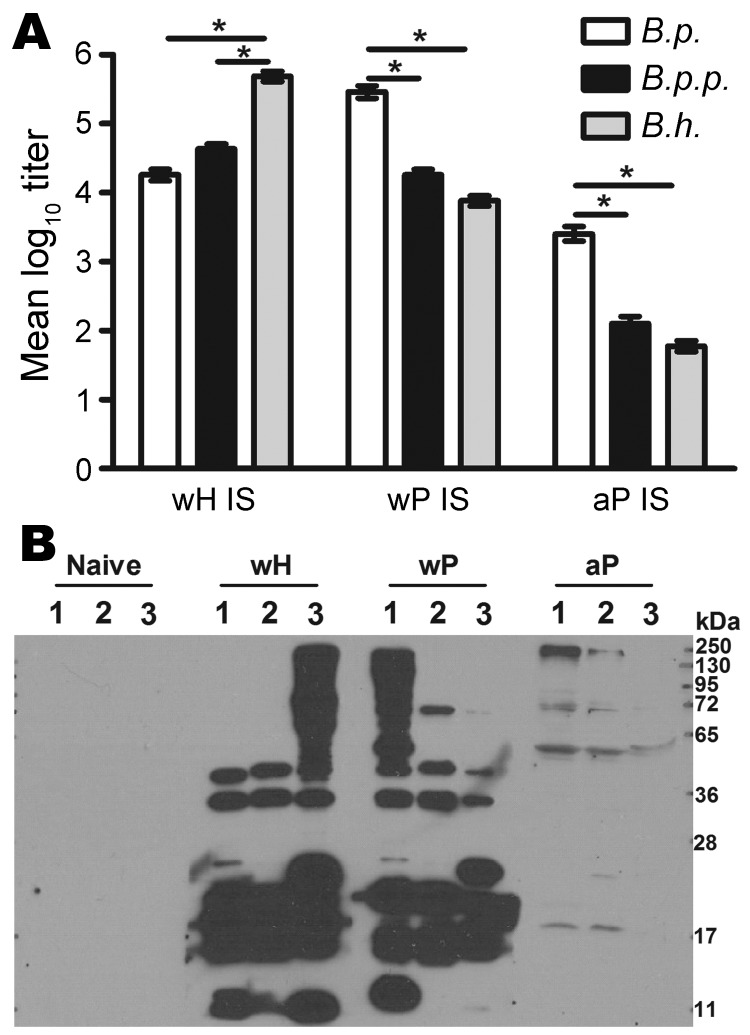
Antibody responses to whole-cell pertussis vaccine (wP), aceullar pertussis vaccine (aP), and whole-cell *Bordetella holmesii* vaccine (wH). A) Specific Ig titers of serum antibodies for *B. pertussis* (*B.p.*), *B. parapertussis* (*B.p.p.*), or *B. holmesii* (*B.h.*) for wH-, wP- or aP- vaccinated mice. Error bars indicate SE. *p<0.01. B) Western blots of *B. pertussis *(1), *B. parapertussis *(2), and *B. holmesii* (3) lysates probed with naive serum or wH-, wP-, or aP-induced serum (IS).

Njamkepo et al. observed that proteins in *B. holmesii* cell lysates are not recognized by antibodies specific for *B. pertussis* vaccine antigens (*14*). To determine whether *B. holmesii* and *B. pertussis* vaccine–induced antibodies are cross-reactive, Ig titers of wH-, wP-, or aP-induced serum samples were examined by using ELISA with heat-inactivated bacteria as antigens. The *B. holmesii*–specific Ig titer of wH-induced serum was >450,000, which is ≈10-fold and 25-fold higher than *B. parapertussis*– and *B. pertussis*–specific serum titers, respectively (Figure 5, panel A); this result indicates partial cross-reactivity. *B. pertussis*–specific Ig titers of wP- and aP-induced serum antibodies were 290,000 and 2,500, respectively. wP- and aP-induced antibodies bind less well to *B. parapertussis* (Figure 5, panel A) and confer little protection against *B. parapertussis* in vivo (*33*). wP- or aP-induced antibodies bound even less well to *B. holmesii*. A similar trend was observed when live bacteria were used to coat the ELISA plates (data not shown), which rules out the possibility that heat inactivation selectively destroys cross-reactive antigens.

To compare the antigens recognized by serum samples from different groups, Western blot analyses were performed on *B. pertussis*, *B. parapertussis*, or *B. holmesii* lysates probed with naive serum or wH-, wP-, or aP-induced serum. wH-induced serum antibodies recognized *B. holmesii* antigens of various molecular masses but lacked cross-recognition of some higher molecular mass *B. pertussis* and *B. parapertussis* antigens (Figure 5, panel B). The wP- and aP-induced serum antibodies bound less efficiently to *B. parapertussis* antigens than to *B. pertussis* antigens, consistent with published data (*33*). Although wP-induced antibodies recognized some *B. holmesii* antigens, they lacked recognition of higher molecular mass (>60 kDa) *B. holmesii* antigens. aP-induced antibodies only poorly recognized a single *B. holmesii* antigen. Together, these data suggest that antibodies generated following *B. pertussis* vaccination do not efficiently recognize *B. holmesii* antigens.

### *B. holmesii*–specific Antibodies and wP-induced Immunity against *B. holmesii*.

On the basis of our data, we discerned that wP induces sufficient T-cell responses but that antibody responses are not sufficient to confer cross-protection against *B. holmesii*. If the lack of antibody cross-recognition is the only reason wP and aP vaccines are ineffective against *B. holmesii* infection, then adding *B. holmesii*–specific antibodies to wP should render the vaccination effective against *B. holmesii*.

**Figure 6:**
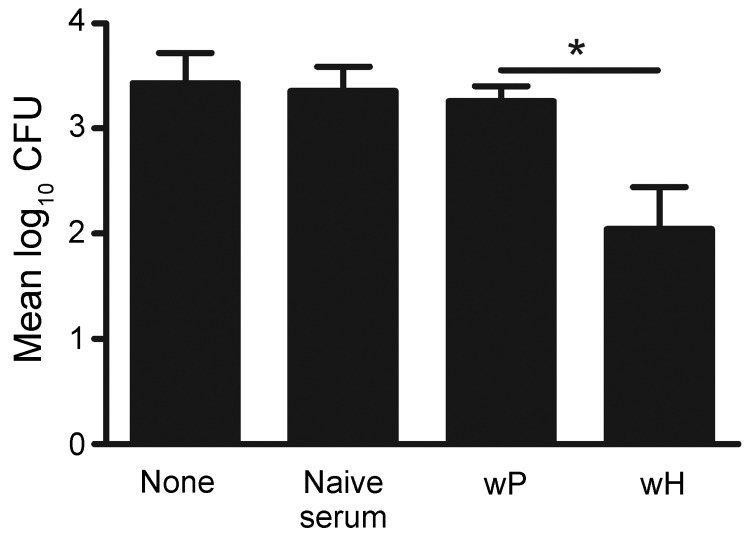
Supplementation of whole-cell pertussis vaccine (wP) with *B. holmesii-* but not *B. pertussis-* specific antibodies. Groups of four wP-vaccinated C57BL/6 mice were left untreated (none) or treated with naive serum, wP-induced sereum, or whole-cell *Bordetella holmesii* vaccine (wH)–induced serum, and challenged with *B. holmesii*. Bacterial numbers in the lungs on day 3 postinoculation are shown. Error bars indicate SE. *p<0.05.

To test this hypothesis, C57BL/6 mice were wP vaccinated or left untreated and later received either naive serum or wP- or wH-vaccinated mouse serum before *B. holmesii* challenge. *B. holmesii* lung colonization was determined 3 days postinoculation. Neither naive serum nor wP-induced serum reduced *B. holmesii* numbers, but vaccinated mice that received *B. holmesii*–immune serum had substantially lowered *B. holmesii* numbers in the lungs (Figure 6). The finding indicates that the addition of *B. holmesii*–specific antibodies to wP increases its efficacy against *B. holmesii*.

## Discussion

The epidemiologic data collected by the MDPH suggest that *B. holmesii* is endemic in Massachusetts and is associated with classic whooping cough–like symptoms (Figure 1). Since the establishment of *B. holmesii* as a species in 1995 (*10*), infections have been sporadically reported worldwide (*6*–*9*,*12*,*13*,*15*,*17*,*18*,*20*,*21*,*23*). Increasing awareness of pertussis and improved analytical tools in industrialized countries may have contributed to the increased numbers of reported *B. holmesii* cases. However, nasopharyngeal *B. holmesii* specimens submitted to the MDPH each year during 2005–2009 likely represent a small fraction of *B. holmesii* infections in Massachusetts. Serologic testing and PCR are the dominant diagnostic *Bordetella* identification assays because of their high sensitivity and time efficiency, but no serologic test or PCR specific for *B. holmesii* is widely accepted or used by the MDPH. Less than 25% of *B. pertussis* cases reported in Massachusetts during 1990–2008 were identified by culture, the only test that currently detects *B. holmesii*. Therefore, larger numbers of *B. holmesii* cases might be identified if additional *B. holmesii*–specific serologic or PCR diagnostic tests are used.

When *B. holmesii* has been clinically identified, several reports have demonstrated little heterogeneity among isolates on the basis of pulsed-field gel electrophoresis banding patterns (*21*) and 16S rRNA heterogeneity (*10*,*17*). By using a sequence-based approach, Diavatopoulos et al. analyzed 7 *B. holmesii* isolates and observed only 1 nonsynonymous polymorphism among 3,666 bases (*16*). Using a similar method, we further analyzed 20 isolates from CDC and 10 from MDPH and identified 174 variable sites among the 2,958 aligned bases; this finding suggests more genetic variation among *B. holmesii* than previously recognized. Similar analyses on a wider range of *B. pertussis*, *B. parapertussis,* and *B. holmesii* isolates could better elucidate the evolutionary history among these human-adapted bordetellae.

Although vaccine studies are better completed in a natural host of the pathogen, the murine model of human-adapted bordetellae infection is well-established. *B. pertussis* and *B. parapertussis* murine infections mimic the course of infection and the immune responses in humans (*30*,*39*,*40*), although an animal model of *B. holmesii* infection has not been previously described. Unlike the classic *Bordetella* species, *B. holmesii* colonizes the murine respiratory tract only when relatively large intranasal inoculums are delivered. This model may be improved, for example, by administering antimicrobial drugs or creating transgenic mice. In this study, reproducible colonization of the respiratory tract was achieved, and statistically significant differences were observed after wH vaccination, which suggests that the mechanisms guiding protective immunity in this model can be used to investigate rapid immune-mediated interactions within the respiratory tract.

We determined that *B. pertussis* vaccines confer little, if any, protection against *B. holmesii*, even though 16S rRNA comparative analysis of *B. holmesii* and *B. pertussis* suggested that *B. holmesii* is closely related to *B. pertussis*. Furthermore, antibodies against *B. pertussis* pertactin, pertussis toxin, fimbriae 2 and 3, adenylate cyclase toxin, and filamentous hemagglutinin recognized few, if any, proteins from multiple *B. holmesii* isolates (*14*), which suggests that these proteins are absent from *B. holmesii* or are antigenically distinct from *B. pertussis*. Although vaccine-induced T-cell responses are cross-reactive to *B. holmesii* (Figure 4), *B. pertussis* vaccine-induced antibodies poorly bind *B. holmesii* (Figure 5). Furthermore, *B. holmesii*–specific, but not *B. pertussis*–specific, antibody administration efficiently decreased *B. holmesii* numbers in the lungs of vaccinated mice (Figure 6), which suggests that the lack of cross-reactive antibody responses may result in poor cross-protection of *B. pertussis* vaccines against *B. holmesii*.

Our data indicate that *B. holmesii* is circulating in Massachusetts and that *B. pertussis* vaccination confers little protection against *B. holmesii*. Careful *B. holmesii* surveillance is required to better evaluate its prevalence and transmission. *B. holmesii* genome sequencing may identify novel virulence determinants to explain its emergence in the human population and guide effective vaccine design.
